# Wild Mallards Have More “Goose-Like” Bills Than Their Ancestors: A Case of Anthropogenic Influence?

**DOI:** 10.1371/journal.pone.0115143

**Published:** 2014-12-16

**Authors:** Pär Söderquist, Joanna Norrström, Johan Elmberg, Matthieu Guillemain, Gunnar Gunnarsson

**Affiliations:** 1 Division of Natural Sciences, Kristianstad University, SE-291 88 Kristianstad, Sweden; 2 Department of Wildlife, Fish and Environmental Studies, Swedish University of Agricultural Sciences, SE-901 83, Umeå, Sweden; 3 Office National de la Chasse et de la Faune Sauvage, CNERA Avifaune Migratrice, La Tour du Valat, Le Sambuc, 13200 Arles, France; University of Lethbridge, Canada

## Abstract

Wild populations of the world’s most common dabbling duck, the mallard (*Anas platyrhynchos*), run the risk of genetic introgression by farmed conspecifics released for hunting purposes. We tested whether bill morphology of free-living birds has changed since large-scale releases of farmed mallards started. Three groups of mallards from Sweden, Norway and Finland were compared: historical wild (before large-scale releases started), present-day wild, and present-day farmed. Higher density of bill lamellae was observed in historical wild mallards (only males). Farmed mallards had wider bills than present-day and historical wild ones. Present-day wild and farmed mallards also had higher and shorter bills than historical wild mallards. Present-day mallards thus tend to have more “goose-like” bills (wider, higher, and shorter) than their ancestors. Our study suggests that surviving released mallards affect morphological traits in wild population by introgression. We discuss how such anthropogenic impact may lead to a maladapted and genetically compromised wild mallard population. Our study system has bearing on other taxa where large-scale releases of conspecifics with ‘alien genes’ may cause a cryptic invasive process that nevertheless has fitness consequences for individual birds.

## Introduction

Anthropogenic impact is one of the biggest threats nature stands before today [1]. One major way in which humans affect biodiversity is by release of alien species [2], [3]. Such exotics, when they become invasive, threaten native fauna through a variety of ways, e.g. competition for resources and predation [4], as well as genetic introgression when they interact with taxonomically close species [5]. Beyond possible effects at the level of ecological communities, there is growing concern about effects on the spatial genetic structure also within species when conspecifics of non-local provenance are released to supplement populations, e.g. for conservation or harvest purposes [6]. A recent review shows that, for a wide range of taxa, reintroduction programs and supplemental stocking of native species need to consider the provenance of the individuals released so as not to introduce non-native genes [7]. Failure to do so may lead to a scenario of ‘cryptic invasion’ *sensu* [8].

The mallard (*Anas platyrhynchos*) is the world’s most abundant duck and one of the most harvested game species globally [9]. It is a well-studied model species in ecology, genetics, epidemiology, game management, and wetland conservation [10]. Large-scale releases of farmed mallards for subsequent harvest by leisure hunting were once popular in North America, but have been largely suspended in favor of habitat (wetland) promotion programs [11]. Still, mallard releases represent a major threat to native populations of other duck species, including rare ones like e.g. Pacific black duck (*Anas superciliosa*) [5], [12], [13]. In Europe, no such problems caused by mallard hybridization with other duck species have been detected, despite annual releases approaching, or even exceeding, numbers comparable to the entire wild population in some countries (e.g. Denmark, Sweden, France, and the Czech Republic). In France alone, more than 1 million unfledged mallards reared in captivity are released each year for hunting purposes [14]. In Sweden the corresponding number is at least 250,000 ducklings (Söderquist, unpublished data). By comparison, the wintering mallard population in France is around 300,000 individuals [15], while the estimated number of breeding birds in Sweden is 200,000 pairs [16].

Given the number of released birds relative to the wild population, the mallard is thus well suited to study general genetic consequences of supplemental stocking. When animals are bred and raised in captivity there is always a risk for phenotypic or genotypic change, which may make released individuals different from wild conspecifics. Founder effects, inbreeding, genetic drift, and selection are the most important mechanisms that may lead to genotypic change in animals kept in captivity [17]. Artificial selection is a way for breeders to influence or maintain the traits of captive individuals, to prevent genetic drift from the wild phenotype and hence raise wild-type individuals only. Besides such deliberate selection of some traits, relaxation of natural selection may also occur in duck farms. For example, behaviors that are crucial in the wild may be altered if non-necessary in captivity. Illustrative examples are predator avoidance, shelter-seeking, certain social interactions, and feeding, which are generally less constraining in captivity [17]. Changed behaviors recorded in captive mallards include habituation to humans [18], sexual behavior [19], and mate preferences [20], [21]. Also morphological changes have been recorded in ducks raised in captivity, e.g. reduced brain volume [22] and altered digestive organs [23], [24].

Feeding in mallard and other dabbling ducks comprises several complex behavioral and morphological mechanisms during which water is sucked in through the anterior opening of the bill, flows through the mandible and maxilla, and food particles are eventually sieved out by the maxillary lamellae as water and detritus are expelled [25]. The density of bill lamellae hence largely determines the minimum size of food particles a duck can obtain by sieving water; the higher the lamellar density the finer particles can be ingested [26]. However, excessively fine lamellar spacing increases the risk of these getting clogged by detritus and mud, which impairs filtering ability [27], [28] cf. [29]. The actual lamellar density in wild populations is therefore the result of a trade-off selection process related to availability, profitability and size distribution of food items [26]. Indeed, Champagnon *et al*. [30] found that lamellar density in the proximate centimeter of the bill was 10% lower in farmed French mallards compared to wild. In addition, Greenwood [31] as well as Pehrsson [32] found that bills of farmed mallards were relatively shorter and wider than those of their wild conspecifics. Due to its crucial function in foraging and feeding, bill morphology can be hypothesized to have important fitness consequences for farmed mallards once released into the wild, and also for the free-living mallard population through potential effects of introgression by farmed stock.

A small proportion of the released ducks survive the hunting and winter seasons and enter the wild breeding population [24]. This leads to an influx of genetic material from farmed mallard stock into the wild. This is not only a local process, though; in Fennoscandia some of the released mallards stem directly or indirectly from Central European stock, translating into a possible spatial reshuffling of the species’ ‘genetic landscape’ at a continental level. Without making any value judgment, mallard releases in Europe may constitute the largest long-term anthropogenic manipulation of a migratory non-fish vertebrate anywhere. As mallards are known to potentially develop altered bill morphology in captivity and because so many have been released for such a long time, it is important to determine if release activity can be correlated to a changed bill morphology in present-day wild mallards over a wider geographic area.

We argue that the mallard study system can serve as a model of general interest, for example by giving insight into consequences of releasing alien genetic material in species that are more difficult to study due to technical or ethical hurdles.

To test the hypothesis that an influx of non-native genes leads to morphological change in the wild population we here compare bill morphology in present-day wild, present-day farmed, and ‘historical pre-release’ mallards from Sweden, Norway and Finland. An earlier test of this hypothesis was restricted to France, a country where massive releases are practiced [30]. The present study considers a much wider geographic area, not being restricted to the release area *per se* (i.e. Sweden) but also including neighboring countries (i.e. Norway and Finland), where releases are not practiced. While the study by Champagnon et al. [30] was useful in demonstrating potential genetic introgression of the wild population by genes from captivity, the present analysis is based on more morphological traits and aims at assessing how widespread the problem potentially is.

## Materials and Methods

### Ethics statement

No approval from the Swedish Animal Ethics Board is needed to trap and ring birds in Sweden. However, national ringing licenses were obtained by the Swedish Museum of National History in Stockholm (license number 632). Strict protocols were followed during all steps where live birds were handled in the study to ensure the safety of the animals. Handling time was kept to a minimum when live birds were measured and no animals were killed specifically for this study. No other specific permissions were needed, for locations or activities, to perform this study. Hunters who donated mallards had hunting permits issued by the Swedish Environmental Protection Agency. All hunts were carried out on private lands with permission from the land owners.

### Sampled birds

Bill morphology was studied in 384 mallards originating from Sweden, Norway and Finland and belonging to three groups; ‘historical wild’ from museum collections, ‘present-day wild’ and ‘present-day hand-reared’ (farmed) (see Table 1 for a break-up of samples by group and country). All museum specimens, both historical and present-day, are adult mallards collected during the breeding season. Historical mallards were collected before 1971, and are thus considered truly wild as the practice of releasing farmed mallards had not begun at the time in northern Europe (Table 1). The wild present-day mallards studied in Skåne, Sweden, were caught in the beginning of the hunting season on a location (WGS84, 56°26′24.2′′N, 13°59′32.5′′E) where farmed mallards had been released in previous years; however in 2011–2012 all farmed mallards released on the location were ringed and thus possible to tell apart from wild birds. Mallards provided from hunts in Dalarna, Sweden, were shot during hunting season before mallard fall migration started and where no releases of mallards occurred. In neither Norway nor Finland have ever any large-scale releases occurred. Because hatch month was known for all farmed mallards we could be certain that all studied specimens were older than 90 days, at which time the bill is fully grown [33]. Our general temporal limitation with respect to sampling month was a means to make sure that the study concerns the Fennoscandian breeding population rather than a mixture of local birds, transient migrants and winter visitors.

**Table 1:** Group, geographic origin, time period, source, and sample size (N) of mallards in this study.

	From year	Source	Sample size
**Historical wild**	1831–1970		**102**
Stockholm	1831–1946	The Swedish Museum of Natural History	22
Uppsala	1856–1934	The Museum of Evolution	17
Gothenburg	1915–1934	The Natural History Museum	24
Jönköping	1903–1920	The Bird Museum	6
Lund	1835–1929	The Museum of Zoology	4
**Sweden total**	1831–1946		**73**
Tromsö	1880–1970	Tromsø University Museum	9
**Norway total**	1880–1970		**9**
Helsinki	1848–1943	The Natural History Museum	19
Kuopio	1899	Kuopio Natural History Museum	1
**Finland total**	1848–1943		**20**
**Present-day wild**	2003–2012		**89**
Dalarna	2012	Hunts	33
Skåne	2012	Trapped alive	18
**Sweden total**	2012		**51**
Tromsö	2003–2010	Tromsø University Museum	36
**Norway total**	2003–2010		**36**
Helsinki	2004–2005	The Natural History Museum	2
**Finland total**	2004–2005		**2**
**Farmed**	2011–2012		**193**
Farm 1 Skåne	2011	Hunts	9
Farm 2 Skåne	2011–2012	At the farm	135
Farm 3 Skåne	2012	Trapped alive	49
**Sweden total**	2011–2012		**193**
**Total**			**384**

### Bill measurements

**Figure 1:**
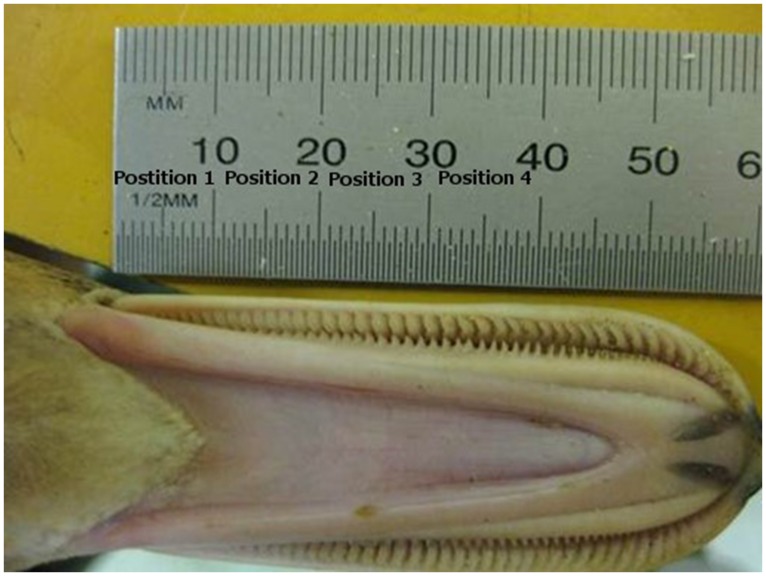
Measurement of bill lamellar density. Scaled photography of the ventral view of a mallard bill used to count maxillar lamellae per centimeter. Positions 1–4 correspond to the first (i.e. proximate, here to the left) four centimeters of the bill. Photograph: Pär Söderquist.

A photograph was taken of the underside of the bill of each specimen. A ruler was placed alongside the bill for scale reference (Fig. 1). The proximate part of the bill was divided into 1 cm segments (hereafter called ‘positions’). For each position the number of lamellae was counted (sample sizes of readings for each position in Table 2). All photographs were analyzed by the same person (JN).

**Table 2:** Sample sizes of studied mallards, broken up by group, country, and sex, used in statistical analyses of bill morphology.

	Bill measurements						Lamellar density							
	Bill height		Bill width		Bill length		Pos. 1		Pos. 2		Pos. 3		Pos. 4	
	♂	♀	♂	♀	♂	♀	♂	♀	♂	♀	♂	♀	♂	♀
**Historical wild**	**60**	**35**	**61**	**38**	**63**	**39**	**28**	**17**	**28**	**17**	**27**	**16**	**26**	**14**
Sweden	49	20	50	20	52	21	21	9	21	9	20	8	20	8
Norway	4	5	4	5	4	5	2	3	2	3	2	3	2	2
Finland	7	10	7	13	7	13	5	5	5	5	5	5	4	4
**Present-day wild**	**55**	**31**	**54**	**30**	**57**	**32**	**38**	**24**	**38**	**24**	**38**	**24**	**38**	**23**
Sweden	28	23	28	23	28	23	26	22	26	22	26	22	26	22
Norway	25	8	24	7	27	9	10	2	10	2	10	2	10	1
Finland	2	0	2	0	2	0	2	0	2	0	2	0	2	0
**Farmed**	**131**	**62**	**131**	**62**	**131**	**62**	**49**	**39**	**49**	**39**	**49**	**39**	**48**	**39**
Sweden	131	62	131	62	131	62	49	39	49	39	49	39	48	39
**Total**	**246**	**128**	**246**	**130**	**251**	**133**	**115**	**80**	**115**	**80**	**114**	**79**	**112**	**76**
	**374**		**376**		**384**		**195**		**195**		**193**		**188**	

“Pos.” means position, which is the sequential number of centimeters proximately in the maxilla.

**Figure 2:**
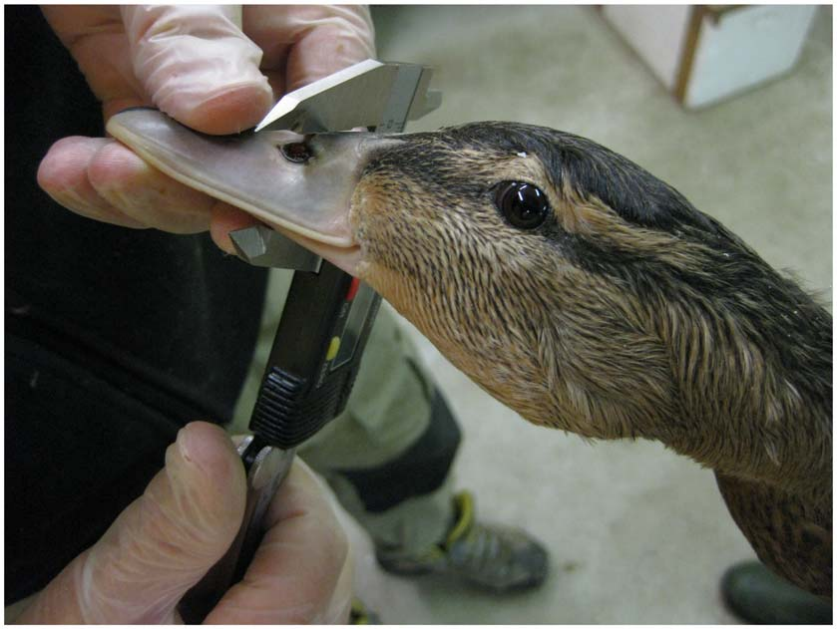
Measurement of bill height and width. Bill height and width were measured (to nearest 0.01 millimeter) over the center of the nostrils with a caliper. Photograph: Pär Söderquist.

**Figure 3:**
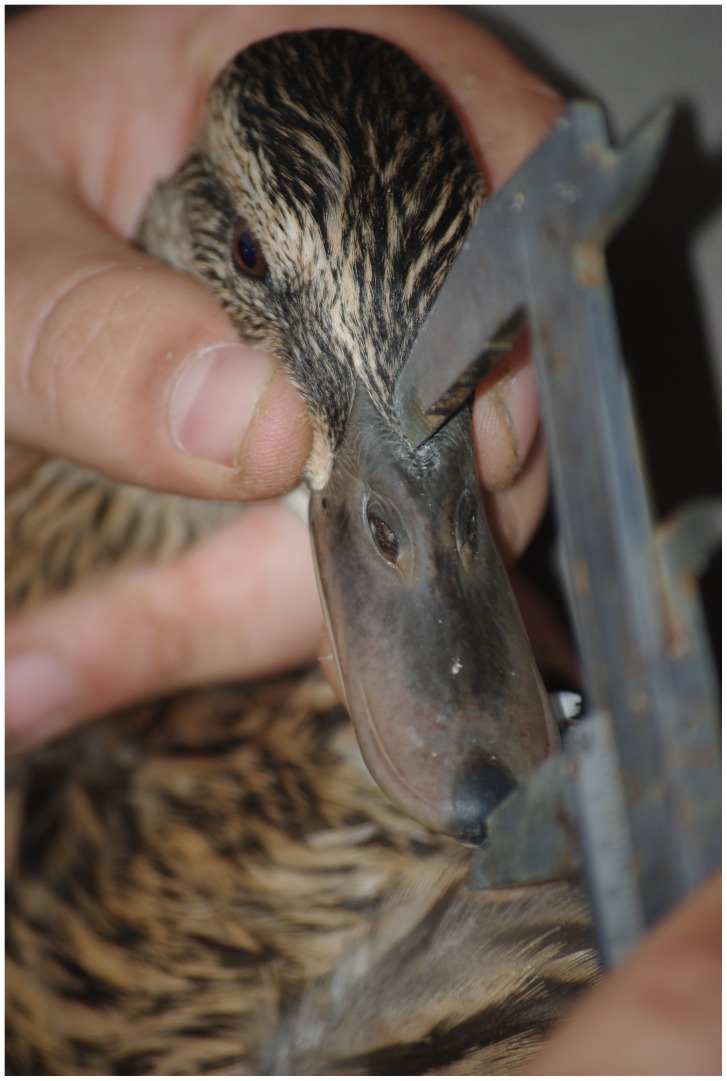
Measurement of bill length. Bill length was measured (to nearest 0.01 millimeter) along the dorsal side of the maxilla using a caliper. Photograph: Pär Söderquist.

Data on bill height, width and length were also obtained from each bird using a caliper measuring to the nearest 0.01 mm. Height and width were measured over the center of the nostrils (Fig. 2). Length was measured along the maxilla, from the tip of the bill to where the feathering begins, i.e. culmen length (Fig. 3). All measurements were made by the same person (PS). Sample sizes differ between subsequent statistical tests because it was not possible to obtain all measurements from some museum specimens, or because some were not photographed (Table 2). Soft parts of museum specimens tend to shrink due to desiccation [34]. Therefore, we corrected all our bill measurements in museum samples by 1.6%, the shrinkage rate previously recorded in mallard [30].

### Statistical analyses

We used IBM SPSS Statistics version 20 and its univariate general linear models (GLM) to test whether bill measurements as well as lamellar density differed between sexes, and between sampling groups (historical wild, present-day wild, and farmed mallards) for a given sex. To contrast groups for significant model outcomes, pairwise Tukey’s post-hoc tests were used. A significance level of 0.05 was used in all tests.

## Results

### Bill measurements

There were significant differences in lamellar density between the sexes for each position, females always having denser lamellae than males (all r^2^>0.068, all *F*_1_ values >15.065, all *p*<0.001) (Table 3). When males and females were considered separately, there was an effect of group within males in position 2 (r^2^ = 0.052, *F*_2_ = 4.133, *p* = 0.019). Post-hoc tests revealed that historical wild males had significantly higher lamellar density than farmed mallards (*p* = 0.020), historical wild mallards also tended to have a higher lamellar density than present-day wild mallards for the same position (*p = *0.051). No significant differences were found in any other position in either sex (*p*>0.116) (Table 3).

**Table 3:** Average number of lamellae (±1 standard deviation) per position ( = 1 centimeter) by group and sex in mallards.

Group	Position 1	Position 2	Position 3	Position 4
**Historical wild ♂**	11.11±1.0^a^	7.94±0.89^a^	6.78±0.63^a^	6.81±0.92^a^
**Present-day wild ♂**	11.13±1.04^a^	7.47±0.69^ab^	6.95±0.73^a^	6.79±0.74^a^
**Farmed ♂**	10.8±1.02^a^	7.43±0.82^b^	6.82±0.7^a^	6.81±0.67^a^
**Historical wild ♀**	12.04±0.82^a^	7.99±0.69^a^	7.07±0.82^a^	7.66±0.88^a^
**Present-day wild ♀**	12.42±1.1^a^	8.29±0.69^a^	7.42±0.72^a^	7.74±0.86^a^
**Farmed ♀**	12.13±1.38^a^	8.05±0.92^a^	7.28±0.92^a^	7.33±0.7^a^

Position 1 is in the most proximate part of the maxilla. In position 2, historical males were statistically different from farmed ones, and nearly so (*p = *0.051) also from present-day wild males. See Table 2 for sample sizes. Different letters indicate significant difference of means within each group and sex.

**Figure 4:**
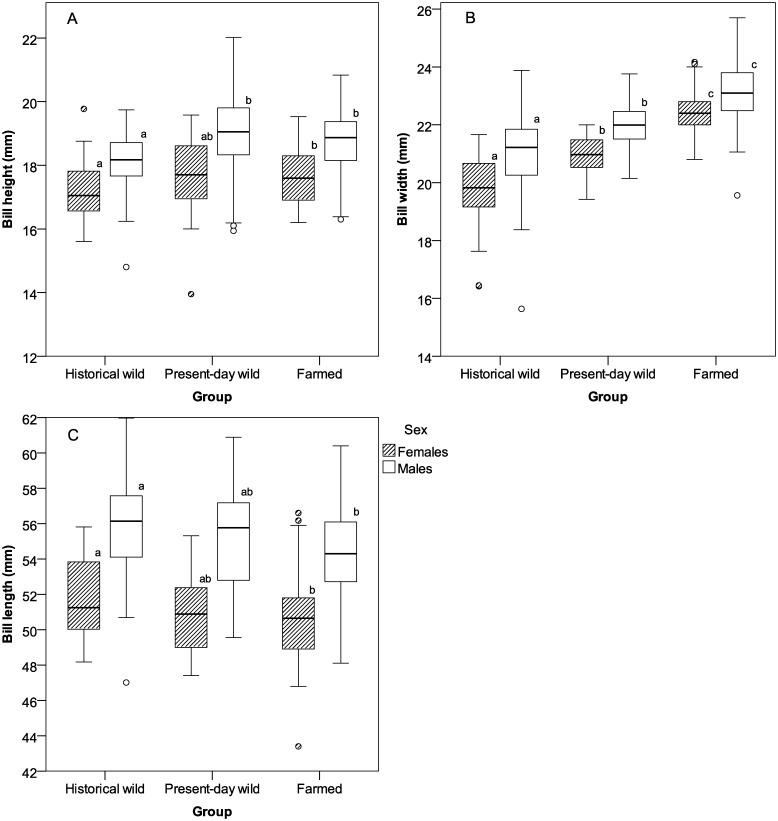
Descriptive statistics of bill measurements. Box plots showing median and quartiles of mallard bill measurements by group and sex; A) height, B) width and C) length, in millimeter. Different letters indicate significant difference of means within each group and sex. Sample sizes for plots are given in Table 2.

Males had higher bills than females (r^2^ = 0.225, *F*_1_ = 109.439, *p*<0.001; Table 4 and Fig. 4A). There was also an effect of group within each sex (males: r^2^ = 0.084, *F*_2_ = 12.286, *p*<0.001; females: r^2^ = 0.041, *F*_2_ = 3.683, *p* = 0.028). Post-hoc tests revealed that historical wild mallards had flatter bills than farmed mallards in both sexes (*p*≤0.036 in both cases). In males, historical mallards also had flatter bills than present-day wild mallards (*p*<0.001) whereas in females there was a tendency for a difference between the two groups (*p*<0.068). No differences in bill height were found between farmed and present-day wild mallards in either sex (*p*>0.451 in both sexes) (Table 4, Fig. 4A).

**Table 4:** Means (±1 standard deviation) of bill measurements (in millimeter) for mallards by group and sex.

Group	Height	Width	Length
**Historical wild ♂**	18.15±0.85^a^	21.15±1.46^a^	55.85±2.64^a^
**Present-day wild ♂**	18.97±1.27^b^	21.89±1.18^b^	55.13±2.67^ab^
**Farmed ♂**	18.79±0.86^b^	23.15±1.03^c^	54.37±2.8^b^
**Historical wild ♀**	17.16±0.96^a^	19.70±1.24^a^	51.88±2.3^a^
**Present-day wild ♀**	17.69±1.19^ab^	20.82±0.9^b^	50.78±2.01^ab^
**Farmed ♀**	17.67±0.8^b^	22.39±0.75^c^	50.63±2.41^b^

See Table 2 for sample sizes. Different letters indicate significant difference of means within each group and sex.

Males had wider bills than females (r^2^ = 0.116, *F*_1_ = 50.288, *p*<0.001; Table 3 and Fig. 4B), but there was also an effect of group within each sex (males: r^2^ = 0.346, *F*_2_ = 65.901, *p*<0.001; females: r^2^ = 0.600, *F*_2_ = 97.685, *p*<0.001). Post-hoc tests showed that farmed mallards had the widest bills and historical wild the narrowest (*p*≤0.003 in all cases) (Table 4, Fig. 4B).

Finally, males also had longer bills than females (r^2^ = 0.327, *F*_1_ = 187.495, *p*<0.001), and there was an effect of group within each sex (males: r^2^ = 0.042, *F*_2_ = 6.419, *p* = 0.002; females: r^2^ = 0.041, *F*_2_ = 3.822, *p* = 0.024). Post-hoc tests showed that historical mallards had longer bills than farmed (*p*≤0.023 in both sexes), while present-day wild were intermediate and could not be separated from any of the two other groups (*p*≥0.113) (Table 4, Fig. 4C).

## Discussion

This study provides evidence that farmed mallards have more “goose-like” bill proportions than do historical and to some extent present-day wild ducks, i.e. they have less lamellae per centimeter, higher, wider, and shorter bills. Our results also show that today’s wild mallards have changed in the direction of farmed mallards since 1971.

### Bill lamellar density

We found a lower lamellar density in the second centimeter of the bill in the Swedish farmed population, but only in males. Champagnon et al. [30] argued that farmed mallards (in France) might have lower lamellar density because they are fed with large food-pellets at the duck farms and therefore the selection for denser lamellae has been relaxed for generations. Also in Sweden, feeding with pellets is a common practice at breeding facilities. Females had higher lamellar density than males (all three sampling groups), which was also reported in Champagnon et al. [30]. This consistent inter-sexual dimorphism can be a cause or an effect of feeding niche divergence in the wild, and an associated reduced inter-sexual competition [35]. In this perspective, it is interesting to note that sexual dimorphism in lamellar density is upheld in farmed birds. In any case, coarser bill lamellae are probably detrimental to mallards in the wild, as this would change the foraging niche by excluding smaller food items such as some seeds and invertebrates [26].

### Bill length, height and width

Males consistently had a bigger bill (i.e. wider, higher and longer) than females, which can be explained by simple allometry as males are larger in general. However, when comparing historical wild, present-day wild and farmed mallards, the pattern is more elusive. Historical mallards of both sexes had longer bills than farmed present-day mallards, a result similar to that reported by Pehrsson [32], who found that farmed mallards had a relatively shorter and wider bill than wild, despite the former being generally bigger [36], [37]. The consequences of such a change are not clear, but studies indicate that some bill morphometrics may be more important than others for which food items the ducks actually collect [38]–[43]. These studies also support the idea that lamellar density affects foraging directly and hence facilitates resource partitioning and species coexistence. However, Nudds and Bowlby [26] did not find any correlation between bill length and the size of ingested food items.

Historical wild mallards had flatter bills than present-day birds (N.B. difference between historical wild and present-day wild females only marginally significant) and historical wild mallards also had the narrowest bills whereas farmed birds had the widest. This is consistent with the results reported by Pehrsson [32] and Greenwood [31]. Regarding differences in bill size, one potential bias needs to be addressed; Champagnon et al. [30] found that bill length in dead (museum) mallards shrank due to drying by 1.6%. The shrinkage pattern may be even more complex since different bill measurements may be affected differently, as reported by Wilson and McCracken [34] for Cinnamon teal (*Anas cyanoptera*). However, a correction term should be species-specific since bill proportions (soft parts as well as bone) may differ substantially between species and thus also the shrinkage patterns [30]. Since the shrinkage pattern reported by Champagnon et al. [30] is the only at hand for mallards, we chose to use this correction (1.6%) for all our bill measurements, acknowledging that bill width and height may be “under-corrected”.

Pehrsson [32] and Champagnon et al. [30] proposed that farmed mallard stock becomes more adapted to feeding on larger food particles on land, such as agricultural crops or food pellets, rather than filtering water for small aquatic seeds and invertebrates. Changed conditions are not only about food item size, though. Food abundance may be a constraining factor in the wild, whereas animals in captivity do not have to worry about finding food. Our study supports this general view, cf. [44], by demonstrating that farmed mallards are different from wild ones and that they have changed in the predicted direction morphologically.

### Conclusions and implications

In short, this study shows that bills of farmed mallards have more “goose-like” proportions compared to historical wild mallards, and that wild mallards in northern Europe have changed in the direction of farmed birds with respect to bill morphology during the time large-scale release programs have been practiced. This change corroborates patterns described by Pehrsson [32] and Greenwood [31]. The present study is also generally consistent with Champagnon et al. [30] in demonstrating a higher lamellar density in the proximate part of the bill in historical wild male mallards, even though the difference was more pronounced in France than in the present study. We speculate this can be due to either a founder effect in the French farming stock or to differences between the study areas in terms of captive conditions (diet, food item size, size-dependent breeding success etc.) The study by Champagnon et al. [30] was also restricted to France, where mallard releases are intensively practiced, while ours also encompass areas with little or no releases (i.e. Finland and Norway). We therefore demonstrate that the gradual diffusion of captive bird characteristics to the wild mallard population is not limited to where the releases occur, but spans over vast geographic areas including release-free zones.

Even though the survival of farmed mallards is low once released into nature [24], the great number of released birds in Europe may lead to introgression of more or less maladapted individuals into the wild population. This process might affect traits evolved under natural and sexual selection in the wild. Moreover, introgression and movement of captive stock between countries could hence promote erosion of large-scale geographic genetic structuring underlying morphological traits cf [45].

A scientific implication of this study is that a deeper understanding is needed of the extent to which shipping of captive stock between countries and survival of released birds have altered natural genetic variation and geographic structuring in European mallards. If farmed stock have unique genetic markers it is valuable to know how abundant such individuals are in different geographic areas, and how well their spatial occurrence correlates with release activity. To increase our knowledge about survival, movements and breeding abilities of farmed mallards, comprehensive individual marking efforts are needed. The pairing process and breeding success of surviving farmed mallards in nature are also essential to study in order to assess the degree of introgression of ‘farmed genes’ into the wild population.

Even though completely wild-like mallards may be nearly impossible to maintain long-term in captivity, there have been several suggestions on how to keep the differences to a minimum. Accordingly, mallard breeders can be encouraged to let the captive stock come in contact with wild birds by using open pens that allow the latter to enter the breeding facility. Secondly, breeders can be encouraged to use local or regional stock instead of importing birds or eggs from other countries. Thirdly, in order to limit drift in bill traits, ducks can be offered feeding conditions that more resemble the natural.

In a larger context, this study exemplifies how releases of conspecifics with a non-native genome can affect wild populations. Cryptic invasions, whether it is by alien species like the common reed (*Phragmites australis*) [8], free-ranging domestic species [46], or farmed birds released for hunting purposes may pose great risks for local wild populations.

## Supporting Information

S1 File **Raw Data.**
(XLSX)
